# Development and Validation of the Pre-Hospital Stroke Symptoms Coping Test

**DOI:** 10.1371/journal.pone.0110022

**Published:** 2014-10-17

**Authors:** Qiuli Zhao, Li Yang, Xiao Zhang, Xuemei Zhu, Qingqing Zuo, Yanni Wu, Liu Yang, Wei Gao, Minghui Li, Shanshan Cheng

**Affiliations:** 1 School of Nursing, The 2nd Affiliated Hospital of Harbin Medical University, Harbin Medical University, Harbin, China; 2 Department of Nursing department, The 2nd Affiliated Hospital of Harbin Medical University, Harbin Medical University, Harbin, China; Azienda Ospedaliero-Universitaria Careggi, Italy

## Abstract

**Background and Purpose:**

Measures of specific knowledge of coping with pre-hospital stroke symptoms can help educate high-risk patients and family caregivers. This study aimed to develop and validate the Pre-hospital Stroke Symptoms Coping Test (PSSCT).

**Materials and Methods:**

Reliability and validity were analyzed using multiple data sources. The Delphi expert consultation method was applied to assess the test’s surface validity and content validity index. The final edition of the 19-item PSSCT contained 3 sections assessing coping with typical symptoms and symptoms associated with vomiting and twitching. Its psychometric properties were investigated in a community sample of 300 high-risk patients and family members.

**Results:**

The PSSCT was readily accepted by participants. It demonstrated adequate surface validity and content validity, and good internal consistency (KR_20_ = 0.822) and test-retest reliability (0.769), with difficulty (P) and degree of differentiation (D) ranges of 0.28–0.83 and 0.15–0.66, respectively. It was also able to distinguish between individuals who had/had not experienced a stroke. Experienced individuals scored significantly higher overall and on coping with typical symptoms and twitching (P<0.01).

**Conclusions:**

The PSSCT can practically and directly assess critical knowledge regarding coping with pre-hospital stroke symptoms and has good reliability and validity.

## Introduction

The global burden of stroke is rapidly increasing. Worldwide, someone is thought to suffer a stroke every other second, with a stroke-related death occurring every six seconds [1]. Thus, strokes are a major cause of death and long-term disability globally, including in developing countries [2]. In China, strokes are also the leading cause of morbidity and mortality [3], with prevalence rates having risen sharply over the past decade. More specifically, approximately 1,500,000 to 2,000,000 new stroke cases are anticipated each year. Its prevalence and mortality rates are estimated to be around 116–219/100,000/year and 58–142/100,000/year, respectively [4], after corrections for age. In order to obtain more satisfactory treatment effects, it is important for patients to find warning signs in a timely manner, adopt the correct pre-hospital coping strategies, and detect and correct inefficient coping methods early; in other words, patients can improve outcomes if they are able to arrive at a specialist or general hospital ward for treatment early on. Several studies have shown that the capacity to identify stroke signs, knowledge of coping with stroke symptoms, and emergency coping abilities during the pre-hospital period are major determinants of successful stroke prevention and treatment [5]–[7]. It is particularly important for family members to have proper knowledge on how to cope with strokes, including their occurrence, prevention, and prognosis [8], [9]. Several studies have demonstrated that the level of knowledge about strokes in the general population is low, which can be improved with the implementation of appropriate health education interventions [10]–[12]. Thus, there is a need for valid assessment tools or evaluations of the effectiveness of stroke education and rehabilitation programs.

A number of tests have been developed to assess specific stroke knowledge, including the Stroke Symptom Questionnaire [13], Stroke Action Test [14], and Stroke Awareness Questionnaire (SAQ) [15], in which residents learn how to dial 911, etc., for general directions when a stroke has occurred. However, there are presently no references to methods assessing community resident knowledge regarding ways to cope with pre-hospital stroke symptoms in the scientific literature. Thus, having an instrument which would allow evaluations of such knowledge among high-risk populations and family members would be a valuable asset to healthcare providers, as it would enable them to more effectively tailor stroke education interventions to help overcome patient concerns. In order to meet the need for a suitable assessment tool, the present study aimed to develop and validate a Chinese test assessing specific knowledge of coping with pre-hospital stroke symptoms among high-risk populations and their family members, known as the Pre-hospital Stroke Symptoms Coping Test (PSSCT). Specifically, our research objectives were to (1) develop the PSSCT and assess its acceptability and feasibility as a tool for evaluating knowledge on coping with pre-hospital stroke symptoms among high-risk populations and their family members, and (2) assess the psychometric properties of the PSSCT.

## Materials and Methods

This experiment was conducted in accordance with the Declaration of Helsinki (1964). All study participants provided informed consent, and the study design was approved by the appropriate ethics review boards.

We used a methodological research design to develop and psychometrically evaluate the PSSCT in this study. The study consisted of two phases, namely (1) the instrument development phase and (2) the evaluation of psychometric properties phase.

### Research Procedure

#### Phase I

Instrument development A total of 25 potential items were originally identified. Of these, twenty were derived from a review of the literature, monographs on stroke knowledge, and the cerebrovascular guidelines for Chinese public health education programs on strokes [4], [16]–[19]. Furthermore, we conducted semi-structured interviews with stroke patients and recorded their answers to these questions. We then extracted a total of five items from the contents of patients’ answers (see **Table 1** for an outline of the interviews).

**Table 1:** Outline of semi-structured interviews.

Interview questions
- How do you cope when stroke symptoms occur?
- Can you talk about how you coped with specific symptoms?

An expert panel of 11 health care providers, including 6 doctors and 5 nurses from the emergency, brain surgery, and neurology departments, was invited to review the items for content, breadth, and applicability, and rate each item on its validity and relevance. The experts were selected according to whether they satisfied the following criteria: (1) possess a high level of training or relevant academic qualifications in the area of cerebral apoplexy (e.g., an associate professor or deputy director of both junior and senior personnel); (2) have a profound interest in and rich clinical knowledge of stroke symptoms, especially with regard to coping with them; (3) have a master’s degree or at least 10 years of clinical experience; and (4) demonstrate a rigorous practical scientific attitude.

The content validity index (CVI) of each item was calculated, and 6 items with indexes <0.70 were subsequently deleted. The 19 items of the PSSCT were finalized after a Delphi expert consultation was conducted. The test was composed of three main parts focusing on coping with typical stroke symptoms and symptoms associated with vomiting and twitching. Dichotomous response options were used. The accuracy of responses, according to guidelines on cerebrovascular care and a review of the literature, was ascertained. A correct answer was given 1 point, and an incorrect one was given 0 points. The total score on the PSSCT reflected the individual’s mastery (i.e., the community resident’s level of knowledge) with regard to coping with pre-hospital stroke symptoms.

#### Phase II

Pilot psychometric testing of the PSSCT The 19-item PSSCT was pilot-tested with 150 individuals at high risk of stroke and 150 of their relatives. Eligible participants were recruited from 3 different communities consecutively across Harbin, China, through posters placed in community publicity columns until the required sample size was reached. The appropriate sample size was determined by the number of participants needed to perform the factor analysis for psychometric assessment of the instrument. As there were 19 potential items, the sample size was calculated based on the subject-to-item ratio of 10–20∶1 [20].

We performed a project analysis of the test (i.e., of item difficulty and degree of differentiation) based on the results of the evaluation in order to validate the effectiveness of the final version of the PSSCT. Its reliability and validity were also assessed. Kuder-Richardson (KR_20_) and test-retest reliability coefficients were used as measures of test reliability, and both content and discriminant validity were evaluated.

### Selection of Research Participants

A sample size of 300 participants was required for this study. The inclusion criteria for 150 high-risk patients coincided with criteria for ascertaining whether individuals were susceptible to stroke: (1) being over 40 years of age; (2) having more than one of several risk factors, including high blood pressure, dyslipidemia (or unknown), diabetes mellitus, atrial fibrillation, and heart valve disease; and (3) clinically diagnosed with cerebral apoplexy (in the past or present).

Meanwhile, the inclusion criterion for 150 of their family members was being a family member of the participant who had close contact with the high-risk population. They could include patients’ children, other family members, friends, or a domestic helper. Exclusion criteria were having mental disabilities or a medical background/education.

### Ethical Considerations

The protocol for this study complies with the Declaration of Helsinki and was approved by the Institutional Review Board of the second affiliated hospital of Harbin Medical University, China. The study was briefly explained to potential participants, who were informed that involvement was completely voluntary and that they could refuse to participate or withdraw from the study at any time without losing benefits or being penalized.

### Statistical Analysis

Descriptive statistics (means and standard deviations) were calculated for the continuous variables, such as descriptions of participants’ clinical-demographic characteristics. The KR_20_ coefficient was used to assess the reliability of the PSSCT. A CVI of ≥0.70 was used as the cut-off to indicate sufficient content validity. We also compared the scores of participants who had and had not experienced a stroke to determine the discriminant validity. A significance level of 0.01 was used for all statistical analyses. All data entry and analyses were performed with the SPSS version 17.0 (SPSS Inc., Chicago, IL, USA), and Epidata 3.0 software, where significance levels were set at a two-tailed p-value of <0.01.

## Results

### Demographic Characteristics of Participants

Three hundred community residents, including patients at high risk of stroke and their family members, were recruited from three local communities to participate in this study. Five pairs of high-risk patients and their family members (n = 10) were excluded, because their knowledge of coping with pre-hospital stroke symptoms could not be evaluated due to logistical reasons. Therefore, 290 (96.7%) valid questionnaires were obtained. The remaining 290 participants (135 males, 155 females; mean age = 62.03 years, SD = 12.69 years) were included in the internal reliability and validity assessments. Most participants (n = 220) had not experienced a stroke, and the remaining 70 had personal experiences with stroke.

The average time taken to complete the original version of the 19-item PSSCT was approximately 5 minutes (no more than 10). The results suggested that the PSSCT had adequate surface validity and was readily accepted by participants. Scores on the test’s 19 items are presented in **Table 2**, according to participants’ characteristics. Total mean scores differed significantly by level of family income per capita, physical condition, living arrangement, health insurance coverage, and smoking habits.

**Table 2:** Scores on 19 items according to participants’ characteristics (n = 290).

Characteristic	N	Participants (%)	Mean Score ± SD	P
**Gender**				P = 0.318
** Male**	135	46.55	11.25±3.47	
** Female**	155	53.45	11.65±3.33	
**Age**				
** <60**	88	30.34	10.90±3.44	P = 0.06
** ≥60**	202	69.66	11.71±3. 36	
**Home address**				
** Urban**	206	71.03	11.71±3.25	P = 0.15
** Suburban**	28	9.66	10.79±3.50	
** Rural**	56	19.31	10.89±3.80	
**Have the disease now**				P = 0.56
** Yes**	218	75.17	11.53±3.34	
** No**	72	24.83	11.26±3.58	
**Living arrangement**				*P = 0.04
** Living alone**	25	8.62	11.68±3.35	
** With family**	248	85.52	11.31±3.41	
** With friends**	17	5.86	13.41±2.76	
**Average family income**				*P = 0.00
** <1000 RMB**	46	15.86	10.02±3.53	
** 1000–2000 RMB**	102	35.17	11.05±3.55	
** >2000 RMB**	142	48.97	12.23±3.04	
**Medical insurance**				*P = 0.03
** Social medical insurance**	175	60.34	11.49±3.20	
** Free medical insurance**	17	5.86	13. 41±2.70	
** Rural cooperative insurance**	65	22.41	10.71±3. 76	
** At one’s own expense**	20	6.90	12.35±3.33	
** Others**	13	4.49	11.00±4.04	
**Physical examination**				
** Many times in a year**	26	8.97	11.77±3.15	*P = 0.00
** Once a year**	83	28.62	11.77±3.14	
** Once every few years**	80	27.59	12.38±3.08	
** Never**	101	34.82	10.42±3.66	
**Smoking**				*P = 0.00
** Yes**	160	55.17	10.55±3.34	
** No**	130	44.83	12.58±3.13	

SD = standard deviation; *P<0.05.

### Analysis of items

Difficulty (P): The difficulty level of a question is typically defined as the proportion of respondents who answer it correctly. Possible values range from 0.0 to 1.0. Items on which more than 90% of respondents answer correctly (i.e., items with a value >0.9) are generally considered to be too easy, while items on which less than 10% of respondents answer correctly (i.e., items with a value <0.1) are considered to be too difficult [21]. In the present study, the analysis revealed that the difficulty (P) of items on the PSSCT ranged from 0.28 to 0.83, as shown in **Table 3**. This range indicated that individual items were answered correctly by 28% to 83% of respondents.

**Table 3:** The CVI, P, and D of each item on the test.

Part	Please judge the appropriateness of the following coping responses in a situation where someone appears to be dizzy and falls suddenly to the ground, showing weakness on one side of the body, numbness, difficulty walking or speaking clearly, or is unconscious or has severe dizziness/headache:	CVI	P	D
**Part I (coping with typical symptoms)**	1. I will call the patient and shake his/her body, checking whether the patient is conscious.	0.85	0.66	0.54
	2. I will place the patient prostrate on the bed, with his/her head raised (on a pillow).	0.90	0.64	0.16
	3. I will pick the patient up from the ground immediately, and check whether he/she is injured.	0.80	0.72	0.62
	4. We should place the patient on our backs, and bring him/her to the ambulance.	0.80	0.79	0.56
	5. We can support the patient with our hands, and bring him/her to the ambulance.	0.85	0.66	0.60
	6. We can observe the patient for a moment; maybe after a moment, he/she will recover.	0.70	0.60	0.50
	7. If the patient has suffered a head injury and is bleeding after falling, we can bandage his/her head to stop the bleeding.	0.90	0.83	0.18
	8. I lack the proper medical knowledge, so I do not give any help to the patient.	0.85	0.28	0.15
	9. We should prevent compression of the patient’s numbness or paralysis of limbs.	0.70	0.87	0.19
	10. We should give the patient nitroglycerin or antihypertensive drugs for lower blood pressure, etc.	0.75	0.29	0.30
	11. We can drop the patient on the bed, and wait for the arrival of emergency personnel.	0.78	0.70	0.66
**Part II (coping with vomiting)**	**Please judge the appropriateness** **of the following coping** **responses in a situation** **where someone appears** **to have the abovementioned** **symptoms accompanied** **by nausea and vomiting:**	**CVI**	**P**	**D**
	12. We should let the patient lie on the bed, with his/her head to one side.	0.90	0.75	0.33
	13. We can flop the patient onto his/her back immediately.	0.75	0.63	0.55
	14. We can give the patient water to eliminate food residues in his\her mouth cavity.	0.85	0.39	0.49
	15. When the patient experiences shortness of breath and has phlegm in his/her throat, we can instruct him/her to cough.	0.70	0.39	0.37
	16. When the patient is vomiting, we can thump the patient’s back, so that we can get the vomit out.	0.72	0.31	0.33
**Part III (coping with twitching)**	**Please judge the appropriateness** **of the following coping responses in** **a situation where someone appears** **to have the abovementioned** **symptoms accompanied** **by twitching:**	**CVI**	**P**	**D**
	17. If the patient is lying on the ground, we should pick the patient up and bring him/her to the hospital quickly.	0.90	0.56	0.65
	18. When the patient twitches, we should turn his/her head to one side, in order to prevent asphyxia.	0.90	0.83	0.25
	19. When the patient twitches and symptoms appear to have disappeared, there is no need to go to the hospital; the patient can have a good rest at home.	0.70	0.70	0.44

CVI = content validity index; P = difficulty (of a test item); D = degree of differentiation (of a test item).

Degree of differentiation (D): An item’s discriminative value indicates how well it distinguishes between high and low scorers. In order to calculate the discriminative value of each item, respondents were first divided into groups of high and low scorers, comprised of the 27% of highest scorers and 27% of lowest scorers. Then, the following formula was used:
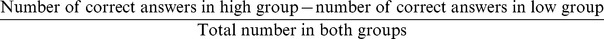



Values of 0.3 or higher commonly indicate (very) good differentiation. Values ranging from 0.15 to 0.3 are indicative of satisfactory to good differentiation, whereas values less than 0.15 are considered poor to mediocre [21]. Our analysis showed that the degree of differentiation (**D**) of items on the PSSCT ranged from 0.15 to 0.66, as shown in **Table 3**.

### PSSCT readability and content validity

To maximize the likelihood that PSSCT items would be easily comprehended by participants, items were written in plain language, and expert reviewers were asked specifically to comment on clarity and assess comprehensibility. The PSSCT was then subjected to participants’ examination; it was administered to the 290 participants with instructions to comment on any difficulties experienced with the items. No difficulties were reported. Thus, the PSSCT showed good readability.

The PSSCT’s KR_20_ was calculated to evaluate its internal consistency, which, according to accepted standards of reliability, was good (KR_20_ = 0.822). The test-retest reliability was also good at 0.769. Through the Delphi expert enquiry method, data on the PSSCT’s content validity were obtained. More specifically, to determine the CVI of each item, the experts were asked to evaluate the relevance of the initial test items with regard to coping with pre-hospital stroke symptoms by using a scale of 1 to 4, where 1 = “absolutely relevant,” 2 = “relevant, but not necessary,” 3 = “a little relevant,” and 4 = “not relevant.” A CVI was then calculated for each item, which reflected the proportion of consulted experts who agreed on the content validity of the item. The results showed that the CVI of test items were all ≥0.7, as shown in **Table 3**.

### Discriminate validity

Significant differences in scores on coping with typical stroke symptoms and coping with twitching were observed between the group of participants who had experienced a stroke and the group who had not. This difference was also evident in participants’ total coping scores (P<0.01). As expected, participants without personal experiences of strokes scored lower than did those with such experience (see **Table 4**), indicating that the PSSCT has good discriminant validity.

**Table 4:** The PSSCT scores of individuals who had experienced a stroke versus those who had not.

PSSCT part	Score (mean ± SD)		P
	With experience n = 70	Without experience n = 220	
**Coping with typical stroke symptoms**	7.14**±**2.01	6.19**±**2.30	*P = 0.001
**Coping with vomiting**	2.54**±**1.29	2.26**±**1.26	P = 0.109
**Coping with twitching**	2.18**±**0.83	1.79**±**0.85	*P = 0.001
**Total coping score**	11.86**±**3.27	10.23**±**3.51	*P = 0.000

SD = standard deviation; *P<0.01.

## Discussion

This is the first report to describe the development and psychometric validation of a Chinese test assessing specific knowledge of coping with pre-hospital stroke symptoms among patients at high risk for stroke and their families. The PSSCT demonstrated good readability and was easily understood by participants, as a majority of them completed the test in 5 minutes. The results indicated that the PSSCT scores of individuals who did not smoke and who underwent annual physical examinations were higher than the scores of those who smoked and did not undergo annual physical examinations. These findings paralleled Yoon and Hickey’s findings [22], [23]. They also found that smoking was the most common risk factor for stroke, and observed that participants who did not smoke and who underwent annual physical examinations scored higher for recognition of stroke risk factors and warning signs. In addition, the highest scores in this study were found among participants who lived with their families and friends. This was consistent with previous reports [15] in which individuals with family support scored noticeably higher than did those with poor family support on knowledge of risk factors for stroke. It is possible that increased levels of social support (including informational support) contributed to higher levels of knowledge in participants; familial responsibilities have also been found to influence the use of health services [24], [25]. In our study, the PSSCT scores of individuals whose family incomes per capita were high and who had free medical insurance were higher than were those of other participants. This has also verified in previous stroke-related knowledge-based surveys [26], [27]. In other words, our study, as well as previous research on identification of stroke risk factors and warning signals, indicates that a greater concern for one’s health (e.g., not smoking and going for regular physical examinations) and stronger support (e.g., greater family support and income, good health insurance coverage) lead to better stroke-related knowledge.

Item analyses allow revisions of tests on the basis of their scores, and are an essential part of improving instructions for respondents [28]. Thus, the difficulty level and discriminative value (degree of differentiation) of each item on the test were analyzed and found to be appropriate. In intervention studies, the validity of measurement/evaluation tools is an important measure of performance [29], as is the difficulty level of a question, often defined in terms of the proportion of respondents who answer it correctly. The PSSCT, a test containing 19 items of varying difficulty levels, was used to evaluate individuals’ cognitions regarding the correct coping responses to stroke symptoms in this study. However, when respondents were tested on their knowledge regarding the onset of stroke symptoms with the items “I lack the proper medical knowledge, so I do not give any help to the patient” and “We should give the patient nitroglycerin or antihypertensive drugs for lower blood pressure, etc.,” only about 28% and 29% of them (i.e., item difficulty levels = 0.28 and 0.29, respectively) were able to recognize the correct coping responses. This worrying result suggests the urgent need to improve the level of coping knowledge with regard to stroke symptoms among high-risk stroke patients and their family members in order to reduce the harm caused by strokes. Thus, designing and implementing comprehensive, professionally organized stroke health education programs aimed at community residents should be a priority.

The primary aim of this study was to develop the PSSCT as an evaluation tool with sound psychometric properties. The KR_20_ coefficient was used in this study to estimate the reliability of the test as a whole and of each of its dimensions through all the stages of its development. The PSSCT yielded an overall KR_20_ coefficient of 0.822. Prior research has indicated that content validity is the most important type of validity because it ensures congruence between the research target and data collection tool [20]. Evidence for the PSSCT’s content validity was based on both a review of the literature and the evaluation of eleven experts through the Delphi expert consultation method. The results demonstrated that all of the instrument’s 19 items met acceptable CVI values of 0.70 or greater [30].

In this study, the PSSCT scores of individuals who had experienced a stroke were higher than were those of individuals who had not, which coincided with previous findings [31]–[33]. Our analysis revealed that those with experience had significantly higher total coping scores than did those without experience. Individuals who had experienced a stroke also scored significantly higher on coping with typical stroke symptoms and coping with twitching. This was likely the result of various factors. First, participants with such experience had been at the scene of the onset of a stroke or had personally encountered a stroke. They also tended to remember the stories of others they had met who had also experienced a stroke. Second, the caregivers of individuals who had experienced a stroke were usually long term. These caregivers were also typically responsible for managing not only the patients’ daily lives but also their disease-related issues [34], [35]. For example, caregivers were often very active in gathering information/knowledge about strokes. However, no significant differences in mean scores for coping with vomiting were observed between the two groups of participants in this study. Furthermore, when they were assessed on their knowledge pertaining to coping with the onset of symptoms associated with vomiting (e.g., “We can give the patient water to eliminate food residues in his\her mouth cavity;” “When the patient experiences shortness of breath and has phlegm in his/her throat, we can instruct him/her to cough;” and “When the patient is vomiting, we can thump the patient’s back so that we can get the vomit out”), only about 30% to 40% of the residents knew how to respond correctly. A possible explanation for this discrepancy is that the occurrence of vomiting is relatively common and one that people tend to ignore. On the other hand, it might be an indication of the lack of knowledge residents have on how to cope with symptoms.

In addition to the abovementioned robust psychometric evidence, data and information presented in the section 3 provided support for the ability of PSSCT scores to reflect individuals’ knowledge pertaining to coping with pre-hospital stroke symptoms. Several studies have shown that community residents lack stroke-related knowledge [12], [19], [22], including knowledge regarding risk factors for stroke and stroke warning signs, and awareness of strokes and transient ischemic attacks. What remains less clear is the extent of their knowledge regarding coping with stroke symptoms. Thus, the present study was conducted to fill this gap in understanding.

As with other instruments, there were several limitations to the PSSCT. The PSSCT can replicate only a part of what would actually be experienced by community residents in a real stroke situation. Therefore, continued efforts to portray symptoms in a more realistic manner are necessary. For example, multimedia technology (e.g., animation technology or vivid pictures) might be utilized to further increase the predictive value of test scores. Second, the purpose of this study was to develop and validate the PSSCT and not to conduct an epidemiological investigation. The participants recruited into the study might not have been representative of China’s population at large. Therefore, caution is needed when generalizing the results of this study to other situations. Finally, a factor analysis was not performed in this study. Further examinations of the instrument’s construct validity are thus required. Recommendations for future research include conducting further evaluations of the instrument in a greater number and wider range of groups, and developing and validating a culturally sensitive English language version of the PSSCT.

## Conclusion

This study aimed to develop a practical 19-item instrument to evaluate knowledge of coping with pre-hospital stroke symptoms among community residents. The PSSCT demonstrated robust internal consistency reliability and validity. Our findings also highlighted the importance of or necessity for community-oriented public education programs on the subject, as many residents lacked essential coping knowledge with regard to pre-hospital stroke symptoms. The insufficient coping knowledge of high-risk populations susceptible to stroke and their family members observed in this study presents a critical problem, but also vital clues into future directions for community health education on stroke. Furthermore, use of a standardized instrument, such as the PSSCT, would greatly facilitate cross-study comparisons.
